# Supplementary material for: Venomix: a simple bioinformatic pipeline for identifying and characterizing toxin gene candidates from transcriptomic data
Source: PeerJ. 2018 Jul 31;6:e5361. doi: 10.7717/peerj.5361 (PMC6074769; doi:10.7717/peerj.5361)
Supplement: Supplemental Information 3 [file peerj-06-5361-s003.gz › FinalOutput_E-20/Zinc_metalloproteinase@disintegrin_5/finaltree.pdf]

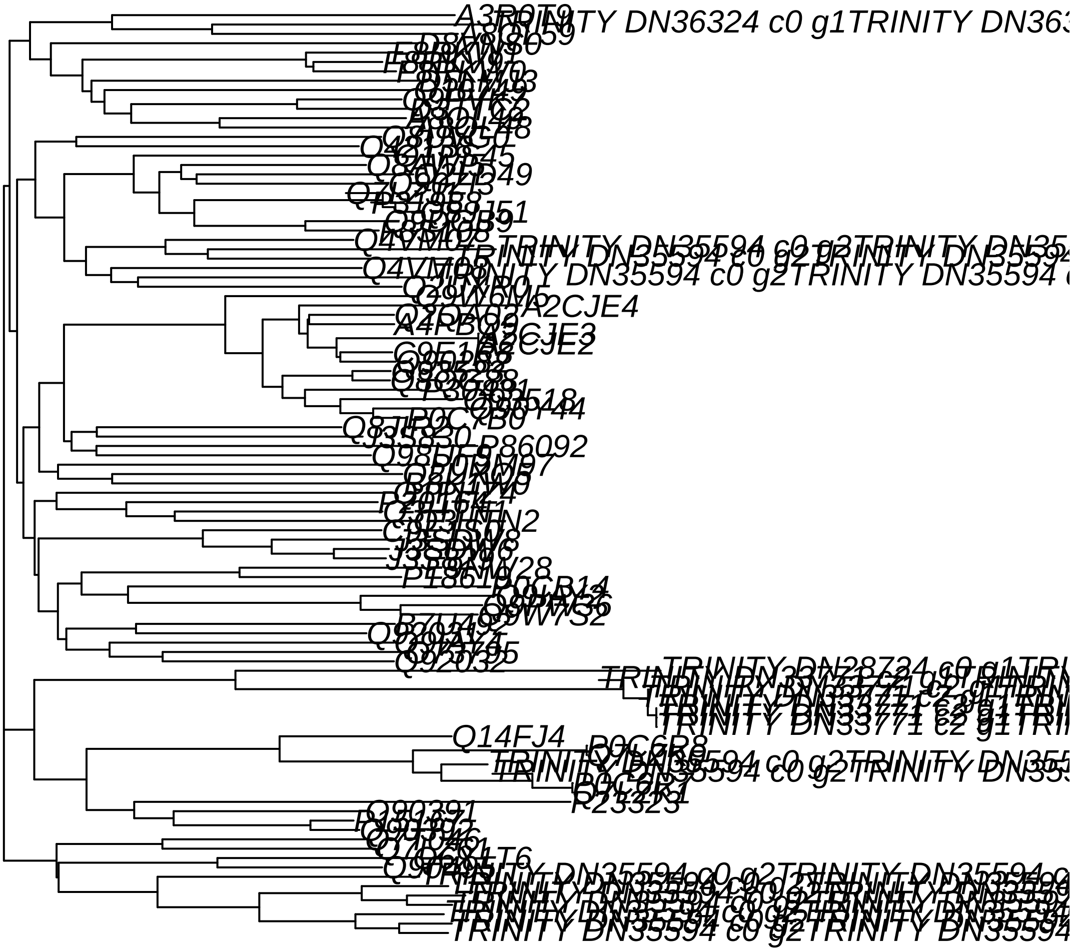

A3R0T9
TRINITY DN36324 c0 g1TRINITY DN363
Q4VM08
Q4VM09
A2CJE4
A2CJE3
A2CJE2
P86092
Q9W7S2
Q92032
Q14FJ4
P0C6R8
P23323
Q90391
TRINITY DN35594 c0 g2TRINITY DN35594
